# AAV2-mediated *in vivo *immune gene therapy of solid tumours

**DOI:** 10.1186/1479-0556-8-8

**Published:** 2010-12-20

**Authors:** Sara A Collins, Alexandra Buhles, Martina F Scallan, Patrick T Harrison, Deirdre M O'Hanlon, Gerald C O'Sullivan, Mark Tangney

**Affiliations:** 1 Cork Cancer Research Centre, Mercy University Hospital and Leslie C. Quick Jnr. Laboratory, University College Cork, Cork, Ireland; 2 Department of Microbiology, University College Cork, Cork, Ireland; 3 Department of Physiology and Biosciences Institute, University College Cork, Cork, Ireland; 4 Department of Surgery, South Infirmary Victoria University Hospital, Cork, Ireland

## Abstract

**Background:**

Many strategies have been adopted to unleash the potential of gene therapy for cancer, involving a wide range of therapeutic genes delivered by various methods. Immune therapy has become one of the major strategies adopted for cancer gene therapy and seeks to stimulate the immune system to target tumour antigens. In this study, the feasibility of AAV2 mediated immunotherapy of growing tumours was examined, in isolation and combined with anti-angiogenic therapy.

**Methods:**

Immune-competent Balb/C or C57 mice bearing subcutaneous JBS fibrosarcoma or Lewis Lung Carcinoma (LLC) tumour xenografts respectively were treated by intra-tumoural administration of AAV2 vector encoding the immune up-regulating cytokine granulocyte macrophage-colony stimulating factor (*GM-CSF*) and the co-stimulatory molecule *B7-1 *to subcutaneous tumours, either alone or in combination with intra-muscular (IM) delivery of AAV2 vector encoding *Nk4 *14 days prior to tumour induction. Tumour growth and survival was monitored for all animals. Cured animals were re-challenged with tumourigenic doses of the original tumour type. *In vivo *cytotoxicity assays were used to investigate establishment of cell-mediated responses in treated animals.

**Results:**

AAV2-mediated GM-CSF, B7-1 treatment resulted in a significant reduction in tumour growth and an increase in survival in both tumour models. Cured animals were resistant to re-challenge, and induction of T cell mediated anti-tumour responses were demonstrated. Adoptive transfer of splenocytes to naïve animals prevented tumour establishment. Systemic production of Nk4 induced by intra-muscular (IM) delivery of *Nk4 *significantly reduced subcutaneous tumour growth. However, combination of Nk4 treatment with GM-CSF, B7-1 therapy reduced the efficacy of the immune therapy.

**Conclusions:**

Overall, this study demonstrates the potential for *in vivo *AAV2 mediated immune gene therapy, and provides data on the inter-relationship between tumour vasculature and immune cell recruitment.

## Introduction

Cancer cells are capable of evading regular immune responses for a number of reasons: they can secrete immunosuppressive factors [1], there can be down-regulation of antigen expression [2,3] or of major histocompatability complex (MHC) molecules [4,5] and also a lack of co-stimulation [6,7]. With the advent of gene therapy as a tool for cancer treatment, immunotherapy-related approaches to stimulate immune responses against cancer cells include the transfer of immune stimulatory genes such as cytokines or costimulatory genes into cancer cells, enhancing antigen presentation through the manipulation of antigen presenting cells (APCs) and genetic vaccination against cancer cell-specific antigens [8,9].

AAV has a number of properties that make it an ideal candidate as a gene delivery vector for the treatment of cancer. AAV elicits only mild host immune responses *in vivo *[10]; long term transgene expression can be achieved [11,12] and also many of the therapeutic genes for cancer treatment fall within the size limit dictated for rAAV. While vectors derived from AAV have shown great promise in the course of research into treatment of numerous indications ranging from cystic fibrosis to haemophilia B [13,14], only in recent years have they begun to be investigated in a cancer setting [15-18].

Granulocyte macrophage colony stimulating factor (GM-CSF) is a cytokine that acts as a critical factor for development and differentiation of macrophages and dendritic cells (DCs). Activation of T cells is enhanced by local GM-CSF mediated recruitment of DCs, allowing for the efficient uptake of antigens and presentation to T cells in the draining lymph node. Co-stimulatory molecules are essential for correct T cell activation and subsequent differentiation into effector T cells following their interaction with antigen presenting cells (APCs). The initial signal for activation is dependent on specific T cell receptor (TCR) recognition of the antigen presented by MHC molecules on APC. The second signal is delivered through the binding of co-stimulatory molecules expressed on the APC surface with their ligands on T cells. A lack of co-stimulatory signals allows tumour cells to induce antigen specific tolerance or anergy on the basis of MHC class I restricted presentation [19,20]. The CD28 receptor has been identified as one of the most important costimulatory receptors on T cells. The ligands for this receptor are members of the B7 family and include B7-1 (CD80) [21,22]. B7-1-transduced tumour cells are expected to present both the antigen and the co-stimulatory (CD28-mediated) signals to CD8^+ ^CTL simultaneously, leading to efficient activation of CTLs without requiring the assistance of CD4^+ ^helper T cells. Transfection/transduction with B7-1 has resulted in tumour cell rejection in several tumour models [19,23-26]. Studies have also demonstrated that cells modified to express GM-CSF or B7-1 can be used to induce protective, T cell-mediated immune responses. Different approaches have been taken for the modification of cells, including both *ex vivo *viral transduction of leukaemia cells [27] and non-viral delivery of the genes on plasmids to growing tumours [28].

For effective cytotoxic responses, in addition to effective education/priming of the immune system to tumour antigens, the local tumour environment must permit immune cell infiltration. Angiogenesis is the formation of new capillary blood vessels from existing microvessels which occurs in physiological and pathological states [29]. This process is controlled by numerous angiogenic factors that are able to attract endothelial cells from the surrounding tissues and represents a crucial stage in tumour growth and metastasis [29,30]. For cancer therapy, strategies based on the manipulation of angiogenesis are referred to as anti-angiogenic strategies and seek to prevent new vessel formation or to inactivate pre-existing vessels. Although angiogenesis is a discrete component of the tumour phenotype, it is often neglected by tumour immunologists. However, lymphocyte extravasation is tightly controlled by blood vessels and requires orchestration of multiple receptor-ligand interactions as well a favourable cytokine/chemokine micromilieu [31]. Moreover, ongoing angiogenesis induces profound morphological and molecular changes in tumour blood vessels and may thus contribute significantly to the tumour's intrinsic resistance to infiltration by immune cells. Therefore, effective tumour immune strategies require both fully armed effector cells and a tumour environment permissive for infiltration and destruction.

The invasive and metastatic behaviour of tumour cells is regulated by extracellular growth factors like hepatocyte growth factor (HGF), which is a ligand for the c-Met receptor tyrosine kinase [32,33]. HGF is a heterodimeric molecule and functions of HGF include mitogenic, motogenic, morphogenic and anti-apoptotic activities [34,35]. In cancer, HGF stimulates malignant cell invasion behaviour through its binding to c-Met [32,33,36]. Nk4 (also known as IL32b) inhibits HGF-c-Met signalling and therefore tumour metastasis [36,37]. Nk4 also has an additional, independent function, promoting anti-angiogenic activities. This is achieved due to the make up of Nk4, which consists of the N-terminus of HGF, containing an N-terminal hairpin and four kringle domains (well described anti-angiogenic molecules) [38-41]. Nk4 augments anti-angiogenic activities through the competitive inhibition of binding of angiogenic growth factors such as VEGF, bFGF and HGF to endothelial cells by its N-terminus [36,42,43]. Angiogenesis-inhibitory as well as cancer-specific apoptosis inducing effects make the *Nk4 *gene an attractive candidate for gene therapy of cancer.

The aim of this study was to assess AAV2 mediated delivery of the immune stimulating genes *GM-CSF *and *B7-1 *and *Nk4 *on different tumour models *in vivo*. Since immunotherapy has the potential to recruit a systemic immune response against tumour cells, and *Nk4 *treatment is known to inhibit angiogenesis and metastatic spread, a combination of these therapies may improve or replace traditional treatments currently available.

## Materials and methods

### Vector constructs

**Figure 1:**
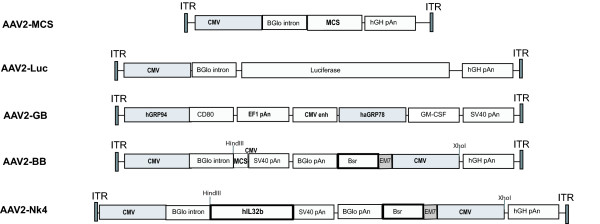
**Vector constructs**. Schematic of coding regions of AAV2 vector constructs used in this study. AAV2-MCS: Cloning construct. AAV2-Luc: Firefly luciferase expressing vector. AAV2-*GB*: Vector encoding both GM-CSF and B7-1 genes. AAV2-BB: BackBone Vector relating to AAV2-*Nk4*, lacking the discrete Nk4 coding sequence but containing all other sequences. AAV2-*Nk4*: Vector encoding Nk4 sequence.

pAAV2-MCS (Stratagene) was used to generate reporter and therapeutic vectors and for the generation of AAV control particles. The mammalian expression vector pVivo1 was purchased from Invivogen (Cayla SAS, Toulouse, France). A version of this plasmid, designated pVivoGMCSF, B7-1, containing the murine *GM-CSF *and murine *B7-1 *genes transcriptionally controlled from two human glucose regulated protein (GRP) promoters GRP94 and hamster GRP78 promoters respectively was designed and cloning was performed on contract by Invivogen. An AAV plasmid encoding the GM-CSF, B7-1 expression cassette (pAAV2-GB) was constructed by excising the expression cassette from pVivoGMCSF, B7-1 using SspI and NheI and cloning the Klenow treated fragment into NcoI and XbaI sites of pAAV-MCS plasmid (Klenow treated). Inserts were confirmed by sequencing (MWG Biotech). The AAV2-*Luc*, AAV2-*Nk4 *and AAV-BB constructs have previously been described [44]. All constructs used in this study are illustrated in Figure 1.

### Vector generation

Recombinant AAV2 vectors (rAAV), AAV2-MCS, AAV2-*GB*, AAV2-*Nk4*, AAV2-BB and AAV2-*Luc *were generated using the AAV Helper-Free System (Stratagene, Agilent, Dublin). rAAV particles were purified using the Virakit AAV Purification Kit (Virapur, San Diego, USA) per manufacturer's instructions. Purified AAV2-*GB *particles were used to transduce HT1080 cells and FACS analysis for B7-1 expression employed to determine the number of transducing units (TU). Purified AAV2-MCS, AAV2-*Nk4*, AAV2-BB and AAV2-*Luc *preparations were titrated using real time PCR to determine the number of genome copies, using primers specific for the CMV promoter (forward: 5' aaatgggcggtaggcgtgta 3', reverse: 5' gatcggtcccggtgtcttct 3') and were synthesized by MWG Biotech, Germany. A fragment of length 124 bp is expected.

### Cell lines and tissue culture

Murine JBS fibrosarcoma tumour cells [28] and murine Lewis Lung Carcinoma cells were maintained in culture at 37°C in a humidified atmosphere of 5% CO2, in Dulbecco's Modified Essential Medium (GIBCO, Invitrogen Corp., Paisley, Scotland) supplemented with 10% iron-supplemented donor calf serum (Sigma Aldrich Ireland, Ireland), 300 μg/ml L-glutamine. Cell densities were determined by visual count using a haemocytometer. Cell viability was confirmed by Trypan Blue Dye Exclusion (Sigma Aldrich Ireland, Ireland) to be > 95% for tumour induction. Human HT1080 fibrosarcoma cells were maintained in culture at 37°C in a humidified atmosphere of 5% CO2, in Eagle Minimum Essential Medium (GIBCO, Invitrogen Corp., Paisley, Scotland) supplemented with 10% iron-supplemented donor calf serum (Sigma Aldrich Ireland, Ireland), 300 μg/ml L-glutamine.

### In vitro transduction

Cells were seeded in a 12-well plate (HT1080 at 2 × 10^5^, JBS at 5 × 10^4 ^cells per well, LLC at 1.5 × 10^5 ^cells per well) in complete medium 24 h before transduction. On the day of transduction, cells were 80% confluent. 9 × 10^8 ^genome copies (GC) of AAV2-*Luc *or 7 × 10^5 ^transducing units (TU) of AAV2-*GB *in a 0.5 ml volume of transduction medium (DMEM, 2% FBS) were added to individual wells. The plates were incubated for 2 h at 37°C, 5% CO_2 _with gentle rocking at 30 min intervals during the incubation. 0.5 ml post infection medium (DMEM, 18% FBS) was added to each well and incubated at 37°C, 5% CO_2 _for a further 24 h.

### Flow-cytometric analysis and ELISA of transduced cells

Cell surface expression of B7-1 was detected by flow cytometry using a FACScan (Becton Dickinson, San Jose, CA) with CD80-specific antibody, clone L307.4 (BD Biosciences UK Ltd, Oxford, UK). Briefly AAV2-*GB *transduced and mock-infected cells were harvested 48 h post transduction. The cells were labelled with the CD80-specific antibody, an isotype control antibody F (ab') 2 Goat Anti Rat IgG: RPe Mouse ADS (Serotec) or unlabeled. 10,000 events were acquired and analyzed for PE fluorescence. PE was measured on the FL2-channel (short band pass 575 nm filter) and plotted against side scatter. Cells without a conjugated antibody and cells with an irrelevant antibody conjugated antibody were used as controls, thereby correcting for background fluorescence.

Production of GM-CSF from JBS cells was quantified by enzyme-linked immunosorbent assay (ELISA) (Quantikine Mouse GM-CSF Immunoassay R&D Systems, Minneapolis, MN). For quantification of GM-CSF production in transduced cells, AAV2-*GB *transduced and untransduced cell supernatant was harvested 48 h post transduction and the assay was carried out as per the manufacturer's protocol.

### Animals and tumour induction

Mice were obtained from Harlan Laboratories (Oxfordshire, England), and kept at a constant room temperature (22°C) with a natural day/night light cycle in a conventional animal colony. Standard laboratory food and water were provided *ad libitum*. Before experiments, mice were afforded an adaptation period of at least 14 days. Female Balb/C or C57Bl/6 mice in good condition, without fungal or other infections, weighing 16-22 g and of 6-8 weeks of age, were included in experiments. For routine tumour induction, 2 × 10^6 ^JBS cells or 5 × 10^5 ^LLC cells suspended in 100 μl of serum free DMEM or were injected subcutaneously (SC) into the flank. Following tumour establishment, tumours were allowed develop and monitored mostly by alternate day measurements in two dimensions using a Verniers Callipers. Tumour volume was calculated according to the formula V = ab^2 ^Π/6, where a, is the longest diameter of the tumour and b is the longest diameter perpendicular to diameter a. From these volumes, tumour growth curves were constructed. In cases of successful treatment, 100 days with no recurrence was considered a cure. In the case of recurrence, the animal was considered incurable and humanely euthanized when the tumour diameter was between 1.5 - 2 cm. Survival time extended from the time of first treatment to 100 days (successful treatments) or to sacrifice (recurrences).

### In vivo gene delivery

All animal experiments were approved by the ethics committee of University College Cork. Mice were randomly divided into experimental groups and subjected to specific experimental protocols. For tumour experiments, mice were treated as soon as the tumour could be reliably injected (tumour diameter = 0.4 cm on average). For quadriceps muscle experiments, a single intramuscular injection was carried out into the right or left thigh of the animal. Mice were anaesthetized during all treatments by intraperitoneal (IP) administration of 200 μg xylazine and 2 mg ketamine. Viral vector particles were administered by direct intratumoural (IT) or intramuscular injection (IM) in a volume of 50 μl 2 × 10^8 ^- 2 × 10^9 ^GC of replication incompetent recombinant AAV2 particles.

### In vivo confirmation of Nk4 gene delivery and expression

Muscle tissue from animals treated by IM injection of AAV2-*Nk4 *and untreated animals was excised at day 3. The muscle tissue was passed through a nylon membrane in order to disassociate the tissue and create a single cell suspension. The cells were precipitated by centrifugation, the DNA and RNA was simultaneously extracted from the cell pellet using the Qiagen Allprep DNA/RNA kit as per the manufacturers protocol. DNA and RNA concentration was determined using the nanodrop. AAV mediated delivery was confirmed by PCR and AAV mediated gene expression was confirmed by rtPCR. The primers were against the Nk4 sequence Forward: 5'CCTCTCTGATGACATGAAGAAG 3', Reverse: 5'TGTCACAAAAGCTCTCCCC 3'. PCR conditions were as follows HotstarTaq Activation 95°C-15 min, Denaturation 94°C-1 min, Annealing 59°C -1 min, Elongation 72°C-1 min. *Nk4 *DNA was detected by PCR in 50 ng of DNA using HotstarTaq Master Mix Kit (Qiagen) in a Mastercycler (Eppendorf,, UK) PCR machine. The PCR products were visualised on a 1% agarose gel. *Nk4 *expression of transduced muscle was confirmed by rtPCR. Extracted RNA was DNAse treated using Ambion DNAfree kit according to manufacturer's instructions. RNA concentration was determined using the nanodrop. Omniscript RT kit (Qiagen) was used to generate cDNA from 100 ng of total RNA in a 20 μl volume according to manufacturer's instructions. The cDNA was diluted to a final volume of 50 μl following cDNA synthesis using DNasefree H_2_O. 5 μl diluted cDNA was PCR amplified using HotstarTaq Master Mix Kit (Qiagen) in a Mastercycler (Eppendorf, UK) PCR machine. The PCR products were then visualised on a 1% agarose gel.

### Luminescence measurements

For *in vitro *experiments, treated cells were analysed for luciferase activity 48 h post transduction using the Luciferase Assay System (Promega MSC, Dublin), as per manufacturer's instructions. Luminescence was measured using the IVIS Imaging System (Xenogen, UK). *In vivo *luciferase activity from tissues was analysed post- transduction as follows: 80 μl of 30 mg/ml firefly luciferin (Biosynth, Basil, Switzerland) was injected intraperitoneally (IP) and intratumourally (IT). Mice were anaesthetised as before. Ten minutes post-luciferin injection, live anaesthetised mice were imaged for 3 min at high sensitivity using the IVIS imaging system (Xenogen, UK).

### In vivo cytotoxicity assay

The development of an immune-mediated anti-tumoural activity following treatment was tested by *in vivo *cytotoxicity assay [45]. The Winn assay was utilised as follows: mice (six/group) received injections of a mixture of JBS cells and splenocytes from either AAV2-*GB *cured mice or naive mice. Splenocytes were taken 100 days post tumour regression from 'cured' mice for use in Winn assays. Splenocytes were mixed with tumour cells and injected SC in a proportion of 50:1 (10^8 ^spleen cells to 2 × 10^6 ^JBS cells). Mice were then monitored on alternate days for tumour development.

### Statistical Analysis

The primary outcome variable of the statistical analyses was the tumour volume in each mouse measured at each time point. The principal explanatory variables were the different treatment groups. Tumour volume was analyzed as continuous. Treatment groups were analyzed as categorical variables. At each time point, a two-sampled t-test was used to compare mean tumour volume within each treatment group depending on the number of groups being compared. Microsoft Excel 11.0 (Microsoft) and GraphPad Prism Version 4.0 (GraphPad Prism Software Inc, San Diego, CA, USA) were used to manage and analyze data. Statistical significance was defined at the standard 5% level. Survival was analysed using a two-sampled Student's t-test assuming equal variances to compare the average number of days survived per group.

## Results

### Validation of vector constructs and gene expression

**Figure 2:**
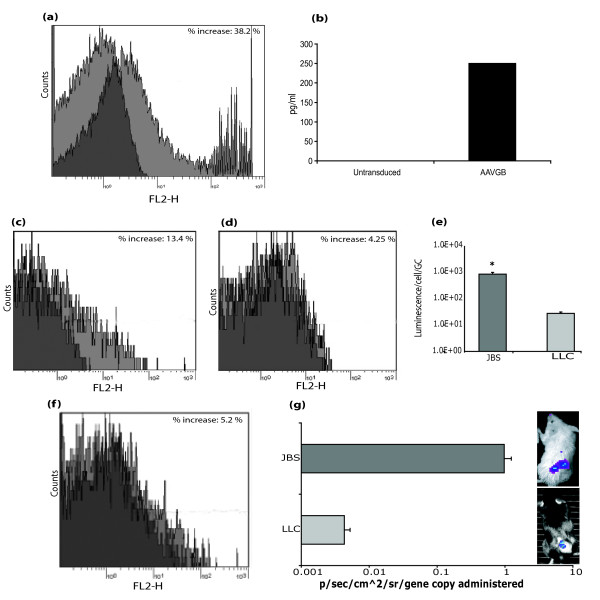
**Validation of immune gene vector construct and transduction efficiency**. (a-b) Gene Expression from AAV2-*GB*. FACS Analysis and ELISA for GM-CSF were used to determine the functionality of AAV2-*GB *particles *in vitro*. A 38.2% (+/- 7.4) increase in B7-1 positive cells was observed in AAV2-*GB *transduced JBS cells. GM-CSF protein was detected in cell culture supernatant in cells transduced with AAV2-*GB *at a level of 250 pg/ml. (c-e) Transduction of JBS and LLC cells *in vitro*. The efficiency of AAV2 mediated transduction of the test cell lines JBS and LLC was determined using FACS analysis for cell surface B7-1 expression following AAV2-*GB *transduction or by luciferase assay following AAV2-Luc transduction. **(c) **A background level of B7-1 expression of approximately 5% was seen in PBS treated JBS cells while a 13.4% (+/- 0.2) increase in B7-1 positive cells was observed in AAV2-*GB *transduced JBS cells. **(d) **A background level of B7-1 expression of 9.4% was observed in PBS treated LLC cells while a 4.25% (+/- 0.15) increase in B7-1 positive cells was observed in AAV2-*GB *transduced LLC cells. **(e) **Luminescence was readily detected in both JBS and LLC cells with a significantly higher level evident in JBS cells (p = 0.004). (* Statistical significance (p < 0.05)). (f) Transduction of LLC *in vivo *with AAV2-*GB*. A background level of B7-1 expression of approximately 10% was seen in PBS treated LLC cells while a 5.2% (+/- 1.48) increase in B7-1 positive cells was observed in AAV2-GB transduced LLC cells. (g) Transduction of JBS and LLC *in vivo *with AAV2-*Luc*. IVIS imaging confirmed AAV transduction of JBS tumours *in vivo *(9.7 × 10^-1 ^p/sec/cm^2^/sr/gene copy administered, +/- 0.27) and LLC tumours (4.3 × 10^-3 ^p/sec/cm^2^/sr/gene copy administered, +/- 0.0009).

Flow Cytometric analysis of cell surface expression of B7-1 and ELISA for GM-CSF confirmed the functionality of AAV2-*GB *particles *in vitro*. The human HT1080 fibrosarcoma cell line was used, being the standard cell line for AAV transduction assays. HT1080 cells were transduced with AAV2-*GB *or mock transduced with PBS. After 48 h, cells and supernatant were harvested for assays. Cells were labelled with anti-CD80 antibody, and the resulting overlay graph (Figure 2a) demonstrated an increase of 38.2% in B7-1 expression in transduced cells (light grey overlay peak) in comparison cells labelled with an isotype (dark grey peak). GM-CSF protein was detected in cell culture supernatant in cells transduced with AAV2-*GB *at a level of 250 pg/ml and not in mock-infected cells (Figure 2b).

The efficiency of AAV2 mediated transduction of each of the test cell lines was determined in cells transduced with either AAV2-*GB *or AAV2-*Luc *particles. FACS analysis for cell surface expression of B7-1 confirmed transduction of both JBS (Figure 2c) and LLC (Figure 2d). These graphs demonstrate that JBS cells (13.4% increase) are more permissive to transduction with AAV2 than LLC cells (4.25% increase). Also evident from these data is that there is a low level of endogenous cell surface B7-1 expression in untreated LLC cells, which has also been reported by other groups [46]. A lower level of background B7-1 expression was observed in JBS cells.

The efficiency of AAV2 mediated transduction of growing JBS and LLC tumours was also assessed. AAV2-*Luc *was administered IT to SC tumours and luciferase expression assessed using the IVIS system on day 7-post administration. Luminescence was detected in JBS tumours in Balb/C mice at 9.7 × 10^-1 ^p/sec/cm^2^/sr/gene copy administered (Figure 2g) and in LLC tumours in C57Bl/6 mice at 1.64 × 10^-8 ^p/sec/cm^2^/sr/gene copy administered (Figure 2g). In order to confirm transgene expression from AAV2-*GB *transduced tumours *in vivo*, LLC tumours were excised 7 days after IT delivery of AAV2-*GB *or PBS. Cell surface expression of B7-1 was detected by flow cytometry as previously described. Results indicated that administration of AAV2-*GB *resulted in an increase in cell surface B7-1 expression. A background level of B7-1 expression of approximately 10% was seen in PBS treated LLC cells while a 5.2% (+/- 1.48) increase in B7-1 positive cells was observed in AAV2-*GB *transduced LLC cells (Figure 2f).

### AAV mediated immune gene therapy of tumours in vivo

**Figure 3:**
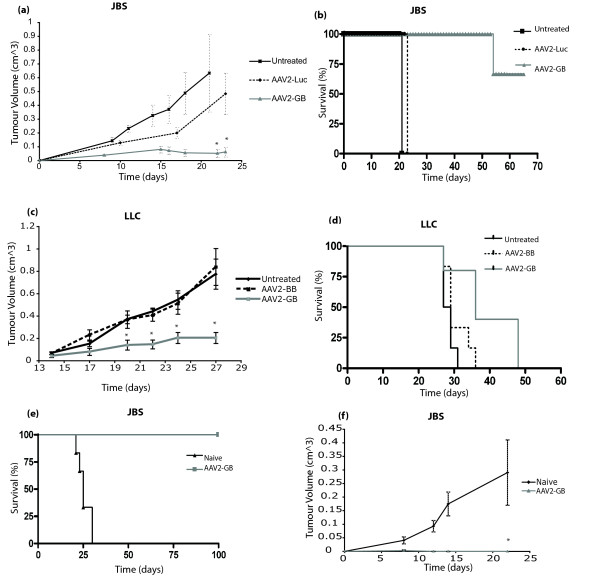
**Immune therapy of tumours *in vivo***. (a-b) Effect of AAV2 delivered *GM-CSF, B7-1 *on SC JBS fibrosarcoma growth *in vivo*. **(a) **Representative growth curve of JBS tumours treated with AAV2-*GB *particles or null vector (AAV2-*Luc*) or untreated. There was a significant difference (p vs. null vector = 0.028, vs. untreated = 0.017) in tumour volume at day 21 between the tumours transduced with AAV2-*GB *and control tumours. **(b) **Approximately 66% animals treated with AAV2-*GB *survived 100 days post treatment, with no signs of tumour recurrence. Treatment with null vector resulted in a slight improvement in survival, but this did not approach significance. (c-d) Effect of AAV2 delivered GM-CSF, B7-1 on SC LLC tumour growth in vivo. Established LLC tumours were treated by IT administration of AAV2-GB or AAV-MCS (control) or no particles (PBS) and growth and survival monitored. **(c) **Tumour volumes in the AAV2-*GB *group were significantly reduced (p < 0.03) when compared with the AAV2-BB administered control group and the untreated group. **(d) **Animal survival in the AAV2-*GB *group was significantly (p = 0.036) increased when compared with the AAV2-*MCS *injected control group and the untreated group. (e-f) Immunological memory following tumour treatment. (e) 'JBS cured' mice (those that had regression of JBS tumour) received a tumourogenic dose of JBS cells on the opposite flank to the original, 'cured' JBS tumour. 100% cured animals receiving JBS displayed no tumour growth, while 0% of JBS naïve controls survived beyond 30 days. **(f) ***In vivo *cytotoxicity assay. Mice received injections of a mixture of JBS cells and splenocytes from either AAV2-*GB *'cured' mice or naïve mice. All mice receiving splenocytes from 'cured' mice failed to grow tumours, while JBS tumours developed in all control animals receiving splenocytes from naïve mice. (* Statistical significance (p < 0.05))

The AAV2-*GB *construct was used to deliver *GM-CSF *and *B7-1 *to JBS or LLC tumours. The JBS study consisted of three groups (n = 5): an AAV2-*GB *treated group, an AAV2 null vector treated group (AAV2-*Luc *vector), and an untreated group. The tumour growth curve (Figure 3a) illustrates a significant decrease in tumour growth rate in those groups treated with AAV particles expressing *GM-CSF *and *B7-1 *genes in comparison with untreated and null vector treatment groups. There was a significant reduction in tumour growth on day 21 between the AAV2-*GB *treated group and the null vector treatment group (p = 0.028), confirming that tumour regression involved the therapeutic genes encoded by the particles and was not due to a response to the particle alone. The survival curve (Figure 3b) illustrates a significant increase in survival for all mice treated with *GM-CSF, B7-1 *in comparison with the untreated controls (p < 0.0008). The treatment resulted in a cure for 60% of the treated animals.

The LLC study consisted of three groups (n = 6): an untreated control group, an AAV null vector (AAV2-BB) group and an AAV2-*GB *treated group. The tumour growth curve (Figure 3c) illustrates a marked decrease in tumour growth in the AAV2-*GB *treated group in comparison with the untreated and null vector controls. The reduction in tumour growth was significant on days 20 - 27 (p < 0.02) between AAV2-*GB *treated mice and both the untreated and null vector groups. There was no significant difference between the untreated and the null vector groups. The survival curve (Figure 3d) illustrated a significant increase in survival in animals treated with *GM-CSF, B7-1 *in comparison with the untreated and null vector groups. Although the increase in survival was significant (p = 0.008), the therapy did not result in cure in any of the treated animals.

### Immunological memory following tumour treatment

In cases where complete tumour regression occurred (60% JBS treated mice), 'cured' mice were rechallenged to assess for sustained anti-tumoural immunological responses. Mice were injected SC on the opposite flank to the original tumour challenge, with tumourigenic doses of the same tumour type (JBS) 30 days following tumour regression. AAV2-*GB *'cured' mice remained tumour free to 100 days whilst all naïve mice developed tumours and were culled due to tumour burden by day 28 (Figure 3e), indicating immunological memory to tumour antigens.

In order to examine for a cell-mediated immune response as a result of AAV2-*GB *treatment, the cytotoxic activity of lymphocytes from treated mice was examined *in vivo *using a modified Winn assay [45]. Groups of mice received injections of a mixture of a tumourigenic dose of JBS cells and splenocytes from either AAV2-*GB *cured mice or naïve mice. All mice receiving splenocytes from 'cured' mice failed to grow tumours, while JBS tumours developed in all control animals receiving splenocytes from naïve mice (Figure 3f), indicating adoptive transfer to naïve mice of anti-tumour lymphocytes conferring resistance to further tumour challenge.

### Nk4 therapy of growing subcutaneous LLC tumours

**Figure 4:**
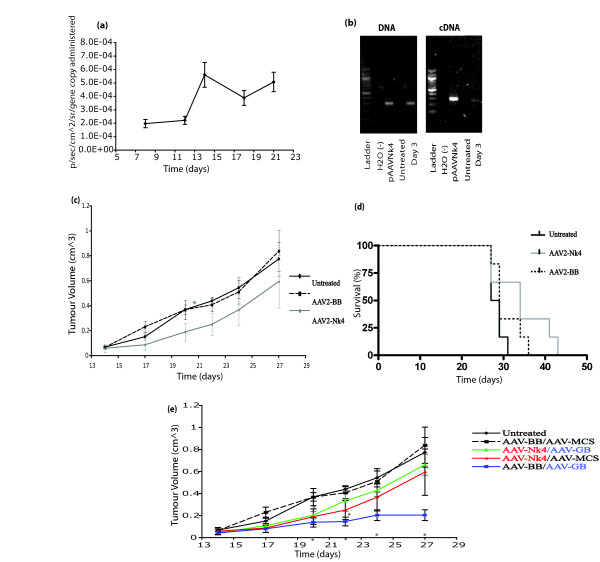
**Nk4 therapy of LLC tumours**. (a) Pattern of AAV-mediated gene expression in muscle tissue. *In vivo *luciferase expression from AAV2-*Luc *transduced muscle tissue was assessed using live whole body imaging (IVIS) at various time-points post delivery. Mean luminescence (p/sec/cm^2/sr) per gene copy ± S.E is shown. (b) Assessment of AAV-mediated *in vivo *delivery and expression of *Nk4*. Quadriceps tissue from animals administered AAV2-*Nk4 *was excised at day 3 and DNA and RNA extracted. *Nk4 *DNA and mRNA was readily detected by PCR in treated muscle tissue confirming AAV mediated delivery and transcription of *Nk4*. No *Nk4 *DNA or mRNA was detected in untreated controls. (c-d) Effect of systemic production of *NK4 *on SC LLC volume. Animals were IM administered AAV-*Nk4 *or AAV2-*BB *(control) or no particles (PBS) 14 days prior to inoculation with LLC tumours. **(c) **Although tumour growth in the AAV2-*Nk4 *group was reduced when compared with the AAV2-BB injected control group and the untreated group at day 27, it proved to be statistically insignificant. **(d) **Although animal survival in the AAV2-*NK4 *group was increased when compared with the AAV2-BB injected control group and the untreated group, it proved to be statistically insignificant (p = 0.26, p = 0.06 respectively). (e) Effect of combined immune gene and Nk4 therapies on LLC tumours. Animals were IM administered AAV2-*Nk4 *or AAV2-BB (control) or no particles (PBS) 14 days prior to induction of SC LLC tumours. Established LLC tumours were then IT administered AAV2-*GB*, AAV2-BB (control) or no particles (PBS) and growth monitored. Although tumour growth in the combined AAV2-*Nk4*/AAV2-*GB *was reduced when compared with the AAV2-BB control group and the untreated group, it proved to be statistically insignificant (p = 0.37, p = 0.51 respectively). (* Statistical significance (p < 0.05))

AAV2 vector mediated transgene expression is known to be delayed initially before increasing over time [47-49]. Given the short therapeutic window available with the fast growing LLC tumour model, and since the Nk4 transgene product is secreted by transduced cells, we opted to administer AAV2-*Nk4 *prior to tumour induction, to quadriceps muscle, with the aim of producing systemic circulating Nk4 protein. The temporal pattern of AAV2-mediated expression from quadriceps was examined using AAV2-*Luc *(Figure 4a). Expression was observed to increase up to day 14 and remain higher thereafter.

For tumour experiments, quadriceps muscles were transduced with AAV2-*Nk4 *14 days prior to LLC tumour induction and tumour growth monitored on alternate days (Figure 4c). Nk4 delivery and expression was confirmed by PCR (Figure 4b). For animals treated with AAV2-*Nk4 *there was a significant reduction in tumour growth compared with the untreated group (p = 0.048 on day 20), although this significance was not seen when compared with the null vector treated group. The Kaplan-Meier curve (Figure 4d) illustrates an increase in survival in animals treated with *Nk4*, although not reaching statistical significance in comparison with the untreated (p = 0.06) or null vector groups (p = 0.26).

### Combination of immune and anti-angiogenic therapies

In an effort to enhance both the immune and Nk4 protocols, a combined therapy was examined. The study consisted of 5 groups: an IT AAV2-*GB*/IM AAV2-BB treated group, an IT AAV2-MCS/IM AAV2-*Nk4 *treated group, a combined IT AAV2-*GB*/IM AAV2-*Nk4 *treated group, an IT AAV2-MCS/IM AAV2-BB treated group (null vector), and an untreated group. The tumour growth curve (Figure 4e) again illustrates a marked decrease in tumour growth in groups treated with AAV expressing *GM-CSF*, *B7-1 *or *Nk4 *genes alone in comparison with the untreated and null vector groups. However, treatment with AAV2-*Nk4 *prior to immune therapy, eliminated the anti-tumour effects of AAV2-*GB *treatment (Figure 4e), with tumour growth in this group similar to controls (p vs. untreated p = 0.52, p vs. null vector control p = 0.38).

## Discussion

Though certain viral vectors can elicit strong immune responses and systemic toxicity [50], gene transduction efficiency is extremely high. While non-viral delivery of plasmid DNA displays lower toxicity, a major obstacle that has prevented its widespread application is its relative inefficiency in gene transfection [51]. The construct used in this study encoded *GM-CSF *and *B7-1*, and was designed such that *GM-CSF *would be secreted from the tumour cell with B7-1 expressed on the cell surface in an effort to elicit an anti-tumour immune response. We have previously demonstrated that non-viral delivery of these immune genes on a plasmid to JBS tumours leads to immune stimulation and consequent eradication of the treated tumour and associated metastases, when delivered by electroporation [28,52] or sonoporation [53].

Other work from our laboratory has shown that AAV2 mediated reporter gene expression in JBS tumours is significantly higher and more sustained than plasmid-mediated delivery. We decided to examine if the anti-tumour efficacy of *GM-CSF, B7-1 *could be improved by AAV2 delivery. The results achieved here were comparable with those observed with both non-viral strategies, suggesting that levels of immune gene expression may not be the limiting factor in recruitment of anti-tumour responses. However, it should be noted that a different temporal pattern of transgene expression is observed with AAV and plasmid vectors. Gene expression from plasmid vector is maximal 24 - 48 h post transfection, while AAV2 related transcription is delayed, taking 4 - 7 days to surpass plasmid levels. Given the relatively short window of therapeutic opportunity permitted by the murine tumour models used here, it is plausible that superior effects might be observed in clinical situations, where patient tumour growth is slower, thereby facilitating AAV vector transcription levels to reach full capacity [8,47-49]. Also, the range of patient tumour locations and sizes amenable to AAV vector administration is far wider than is practical for delivery involving electroporation or sonoporation equipment, which are currently useful only for accessible subcutaneous tumours. Furthermore, the potential for specific transduction of tumour cells following systemic administration of viruses has been validated, involving the inclusion of tumour specific targeting ligands to viral vector surfaces [54,55].

Our studies demonstrated that the observed tumour reductions were immune mediated, and that the immune response induced was, at least in part, T cell mediated. Adoptive transfer of the anti-tumour immune response to naïve animals prevented tumour establishment. The immune gene therapy was less effective in LLC than JBS and this might be attributed to a number of different reasons. Both of the tumour cell lines can be described as weakly or non-immunogenic. The JBS murine fibrosarcoma is derived from the 3T3 cell line and is described previously [56]. JBS grows in immunocompetent Balb/C at the same rate as in athymic mice, and vaccination strategies, using a diversity of approaches, schedules and cell treatments, failed to protect Balb/C mice from subsequent tumourogenic JBS challenges [28,56]. The well characterised LLC cell line has previously been shown to be weakly or non-immunogenic, with a number of approaches such as UV irradiation [57] or viral infection [58] being used to increase their immunogenicity in previous studies. As demonstrated both *in vitro *and *in vivo*, AAV2 transduces JBS cells more efficiently than LLC. The AAV2/2 serotype used in our studies has a reported 30% efficiency of transducing LLC *in vitro *[59]. We observed an even lower efficiency (data not shown). Different serotypes such as AAV2/5 have a 65% transduction efficiency in LLC [59]. Also, as demonstrated here, LLC cells have an endogenous B7-1 expression. It is possible that low level B7-1 expression following transduction with AAV-GB in LLC cells could result in preferential binding of B7-1 to CTLA-4 on Treg cells rather than CD80 on effector cells resulting in a subsequent tumour growth advantage, as demonstrated by Tirapu *et al *[46]. However, such an effect was not apparent in our studies, with the moderate increase in CD80 expression combined with GM-CSF through gene transduction sufficient to significantly reduce tumour growth. An increase in cell surface B7-1 expression breaks the immune tolerance allowing B7-1 to bind preferentially to its main receptor CD28 on antigen presenting cells (rather than Treg) creating an anti-tumour response. We have previously demonstrated that simultaneous depletion of Treg further improves immune therapy [60].

For both tumour types examined here, it is plausible that a minimal threshold of the percentage of tumour cells expressing GM-CSF and B7-1 is necessary for this system to effect complete tumour regression. Intratumoural distribution of transduced cells could also be important. In AAV2 treatment, it is likely that a large portion of the tumour is untransduced, as the transduction region is limited to the needle track. In an attempt to maximise transduction efficiency, we used multiple injections of AAV2 vector in an attempt to saturate the tumour with vector solution. This notwithstanding, it is unlikely that there is a requirement for transduction of every tumour cell, as the mechanism of tumour regression is immune mediated, whereby targeting of non-transduced tumour cells is achieved after the gene therapy induced immune sensitisation.

Due to the nature of the AAV2-*Nk4 *construct, a null vector that encoded the blastocidin antibiotic resistance gene was used as a control. In contrast, the AAV2-*GB *construct includes no extra coding sequences. In this case, the control vector/s used included the AAV2-MCS or AAV2-*Luc*. Firefly luciferase is generally accepted to be non-immunogenic [61], and was used here for monitoring of vector-mediated expression in tumours during experiments. Specifically in relation to JBS tumours, growth curves also illustrate that there is a moderate, but statistically insignificant reduction in tumour growth for those tumours treated with the null vector (AAV2-*Luc*) in comparison with the untreated group. No such tumour reduction in tumour growth was seen in LLC tumours treated with the null vector (AAV2-BB) in comparison with the untreated LLC group.

In this study, we also assessed the effects on tumour growth of combining anti-angiogenic treatment with immune gene therapy. The process of angiogenesis provides an ideal target for treatment, has been studied extensively and anti-angiogenic agents are in clinical use [62]. Solid tumours create a unique microenvironment, featuring chaotic vasculature, resulting in zones of tumour ischaemia and poor intratumoural circulation, which can prevent immune cell access [31,63-65]. In recent years, evidence is accumulating that the immune and vascular environments are closely linked. Several studies have indicated that vascular components of the tumour stroma are targeted during immune-mediated tumour rejection [31]. Furthermore, recent reports indicate that certain anti-angiogenic therapies, rather than eliminating vasculature in the tumour and 'starving' cells, serve to normalise microcirculatory function, permitting access to the tumour for immune cells [63]. By developing a deeper understanding of the activity *in vivo *of various anti-angiogenic agents, new improved therapeutic regimes may be developed by addressing simultaneous anti-angiogenic activity and immune up-regulation, with more therapeutic potential than either therapeutic approach alone. We have previously shown that IT administered AAV2-*Nk4 *did not significantly reduce SC LLC tumour growth [44]. In the current study, systemic production of Nk4 induced prior to tumour establishment provided superior anti-tumour immune responses to IT delivery, with a reduction in tumour burden and an increase in survival in AAV-*Nk4 *treated animals, although differences were not statistically significant. However, the combination studies described here showed that Nk4 therapy eliminated the efficacy of the immune therapy. In our experiments involving prior expression of anti-angiogenic agent, it is possible that Nk4 mediated reduction in tumour neovasculature may act as an obstacle to immune therapy by preventing migration of immune effector cells into established tumour parenchyma [31,64,65]. The schedule and timing of combined treatment may be key in achieving improved responses with such an approach. In addition, since Nk4 is a multifunctional molecule, we cannot rule out the possibility that its activity other than anti-angiogenic, might play a role in the observed responses. The design of the experiment addressed the possibility that pre-exposure to the first AAV vector IM precluded gene transfer with the IT vector one week later due to any potential immune response to the vector. The experimental group that received AAV2-*GB *IT also received AAV-BB IM one week previously, and a significant reduction in tumour growth was still observed, ruling out an immune reaction to AAV preventing expression of the immune genes. Furthermore, as indicated in Figure 4a, IM delivery of AAV2-*Luc *resulted in long-term gene expression indicating the absence of immune responses to AAV-transduced muscle cells.

Much study is required with respect to elucidating the complex cascade of events that is needed to reduce angiogenesis while permitting effector cell entry into tumours. Researching these mechanisms will enable cancer biologists to specifically target the tumour environment and further improve therapeutic efficacies. Together with overcoming the obstacle of inefficient effector cell generation in cancer patients, 'angio-immuno' therapy may provide new opportunities to permit tumour infiltration and destruction. Exploiting the benefits of gene therapy, especially utilising viral vectors such as AAV, in terms of local activity and duration of therapeutic activity, may overcome current obstacles to successful cancer treatment with systemically administered drugs.

## Competing interests

The authors declare that they have no competing interests.

## Authors' contributions

SAC performed the in vitro and in vivo experiments, and contributed to drafting the manuscript. AB constructed AAVNk4, AAVBB. MFS, PTH, GCO'S, DMO'H and MT were the coordinators of the project. MT designed the studies and drafted the manuscript. All authors read and approved the final manuscript.
